# Supplementary material for: Patterns of Intron Gain and Loss in Fungi
Source: PLoS Biol. 2004 Nov 30;2(12):e422. doi: 10.1371/journal.pbio.0020422 (PMC532390; doi:10.1371/journal.pbio.0020422)
Supplement: Table S1 — Also available at http://genes.mit.edu/NielsenEtAl/. (4.3 MB ZIP). [file pbio.0020422.st001.zip › NielsenEtAl/html/327.html]

AN4464.1.NCU02629.1.MG04435.1.FG00969.1


```
 CLUSTAL W (1.82) Multiple Sequence Alignments - Introns Inserted


Sequence 1: NCU02629.1	594 aa
Sequence 2: MG04435.1	595 aa
Sequence 3: FG00969.1	595 aa
Sequence 4: AN4464.1	600 aa
Alignment Length: 601 aa
Number Identitical Residues: 405 aa
Alignment Score (without introns) 17669


MG04435.1 	MASSQRIA~IVSVYDKTGLLDLAKGLIKNNVRILASGGTSKMIRESGFPVE2-----DIS
NCU02629.1	MAANQRIA~IVSVYDKTGLLDLAKGLVQHNVRILASGGTAKMIRESGFPVE2-----DIS
FG00969.1 	MSSQQKIA~IVSVYDKTGLLDLAKGLVQQNVRILASGGTSKMIRESGFPVE2-----DVS
AN4464.1  	-MSQQKSA1ILSVYDKTGLLDLAKGLAKHNVRLLASGGTARMIREAGFPVE2LTDIRDVS
          	  :.*: * *:*************** ::***:******::****:*****  :.  *:*

MG04435.1 	AITKAPEMLAGRVKTLHPAVHAGILARNLESDEKDLAEQSIDKVDYVVCNLYPFKDTVAK
NCU02629.1	AITKAPEMLGGRVKTLHPAVHAGILARELASDEKDLAEQNIEKVDYVICNLYPFKDTVAK
FG00969.1 	AITKAPEMLAGRVKTLHPAVHAGILARDLASDEKDLADQNINKVDYVICNLYPFKDTVAK
AN4464.1  	AITHAPEMLGGRVKTLHPAVHGGILARDIESDEKDLADQNIAKVDFVVCNLYPFKETVNK
          	***:*****.***********.*****:: *******:*.* ***:*:*******:** *

MG04435.1 	INVTVPEAVEEIDIGGVTLIRAAAKNHSRVTILSDPTDYAEFLKELDSGEIKESSRKLYA
NCU02629.1	INVTVPEAVEEIDIGGVTLIRAAAKNHARVTILSDPNDYAEFLKELESGEIKDSSRQLYA
FG00969.1 	INVSIPEAVEEIDIGGVTLIRAAAKNHKRVTILSDPNDYAGFLKELEKGDVTESSRNRYA
AN4464.1  	VNVTIEEAVEEIDIGGVTLLRAAAKNHSRVTILSDPQDYPEFLKELEAGEITETSRRSYA
          	:**:: *************:******* ******** **. *****: *::.::**. **

MG04435.1 	LKAFEHTADYDAAIADFFRKQYASDGLQHLTLRYGANPHQKPAAAFAKEGKLPFKVLCGS
NCU02629.1	LKAFEHTADYDAAISDFFRKQYAGHGLQHLPLRYGANPHQKPAAAYVKEGKLPFTVVNGA
FG00969.1 	LKAFEHTADYDAAISQFFRKEYAGNGDQYSALRYGANPHQKPAAAYVSEGNLPFKVLGGS
AN4464.1  	LKAFEHTADYDTAISGFFRKQYAGNGEKHLSLRYGTNPHQKPASACMPQGKLPFKALNGS
          	***********:**: ****:**..* :: .****:*******:*   :*:***..: *:

MG04435.1 	PGYINLLDALNAWPLVKELKAALGLPAAASFKHVSPAGAAIGTPLSADEQKVYMVEGIEG
NCU02629.1	PGYINLLDAFNAWPLVKELKKALGKPAAASFKHVSPAGAAIGLELTADERKVYMVDDIPG
FG00969.1 	PGYINLLDALNAWPLVKELHKALGKPAAASFKHVSPAGAAIGLPLTEEEKKVYFVHDIEG
AN4464.1  	PGYVNLLDALNAWALVKELKQALGYPAAASFKHVSPAGAAVGVPLSDKERKVYMVDDIAG
          	***:*****:***.*****: *** ***************:*  *: .*:***:*..* *

MG04435.1 	IESSALAQAYARARGADRMSSFGDMIALSDVVDLPTAQIISKEVSDGVIAPGYDAAALEV
NCU02629.1	IETSGLAQAYARARGADRMSSFGDMIALSDVVDVPTATIISKEVSDGVIAPGFEPAAAEI
FG00969.1 	IDESSLAQAYARARGADRMSSFGDMIALSDVVDVPTARIISKEVSDGVIAPGYEEAALEI
AN4464.1  	IESSGLAQAYARARGADRMSSFGDVLALSDVVDVTTAKIISREVSDGVIAPGYEKEALEI
          	*: *.*******************::*******:.** ***:**********::  * *:

MG04435.1 	LKKKKGGRYLVLQMDADYVPSPQELRTVYGVSLAQHRNDVEVSP-ASFSTVLVPKDGPAL
NCU02629.1	LKKKKGGRYLMLQVDPEFEPSKSETRTVFGITLAQGRNDVEISP-ETFSNIITPKDG-TL
FG00969.1 	LKKKKGGRYLVLQIDPEYNPPATETRTVYGINLQQHRNDVEITP-KHFNTIITPKDTASL
AN4464.1  	LSKKKGGKYLVLQMDDSYVPAGEETRTVYGVQLNQHRNDVVISPSKTFNTIITPKDLKTL
          	*.*****:**:**:* .: *.  * ***:*: * * **** ::*:  *..::.***  :*

MG04435.1 	PESALRDLTVATIALKYTQSNSVCYAANGQVIGLGAGQQSRIHCTRLAGDKADNWWLRFH
NCU02629.1	PENAQRDLTIATIAAKYTQSNSVCYAARGQVVGLGAGQQSRIHCTRLAGDKTDNWWLRFN
FG00969.1 	PESAARDLTIATITLKYTQSNSVCYAYNGQVVGLGAGQQSRIHCTRLAGDKADNWWMRFH
AN4464.1  	PDAALRDLTVATIALKYTQSNSVCYALNGQVVGLGAGQQSRIHCTRLAGDKTDNWWMRFH
          	*: * ****:***: *********** .***:*******************:****:**:

MG04435.1 	PRVLGIKWKKGTKRPDKSNAIDLLVSGELPKSGPERESFEALFDEVPAAFGEAEREEWLG
NCU02629.1	PRVIGIKWKKGAKRPDKSNAIDLLVSGQLPKDGPERESFEALFEEVPAAFTEQEKNEWLS
FG00969.1 	ERVLGIKWKKGTKRPDKSNAIDLLVSGQLPKDGPEREAFEGVFEEVPAAFTAEEREAWMK
AN4464.1  	ERVLNIKWKAGTKRADKSNAIDLLCSGQTPRNEIEQAEYERVFEEVPAPFTQEERESWLK
          	 **:.**** *:**.********* **: *:.  *:  :* :*:****.*   *:: *: 

MG04435.1 	KLTNVAISSDAF0FPFTDNVYRAARSGVKYMAAPSGSQNDQPVFDTADKLGITFVEQSVR
NCU02629.1	KLTDVCISSDAF0FPFVDNVYRAARSGVKYIAAPSGSQNDSPVFETAEKLGITFVEQNTR
FG00969.1 	QLKNVCVSSDAF0FPFIDNVFRVSQSGVKYVAAPGGSQNDSAVFDTAEKLGITFVEQNIR
AN4464.1  	QLSEVAVSSDAF0FPFIDNVFRAARSGVKYIAAPSGSQNDGPVFETAEKLGITFVEQGTR
          	:*.:*.:***** *** ***:*.::*****:***.***** .**:**:*********. *

MG04435.1 	LFHH
NCU02629.1	LFHH
FG00969.1 	LFHH
AN4464.1  	LFHH
          	****
```
